# Is the clinical frailty scale feasible to use in an emergency department setting? A mixed methods study

**DOI:** 10.1186/s12873-023-00894-8

**Published:** 2023-10-26

**Authors:** Erika Hörlin, Samia Munir Ehrlington, Rani Toll John, Joakim Henricson, Daniel Wilhelms

**Affiliations:** Department of Emergency Medicine, Department of Biomedical and Clinical Sciences, Linköping University, Linköping, Sweden

**Keywords:** Clinical frailty scale, Feasibility, Frailty, Implementation, Emergency medicine, Geriatric medicine, Mixed methods

## Abstract

**Background:**

The Clinical Frailty Scale (CFS) is a frailty assessment tool used to identify frailty in older patients visiting the emergency department (ED). However, the current understanding of how it is used and accepted in ED clinical practice is limited. This study aimed to assess the feasibility of CFS in an ED setting.

**Methods:**

This was a prospective, mixed methods study conducted in three Swedish EDs where CFS had recently been introduced. We examined the completion rate of CFS assessments in relation to patient- and organisational factors. A survey on staff experience of using CFS was also conducted. All quantitative data were analysed descriptively, while free text comments underwent a qualitative content analysis.

**Results:**

A total of 4235 visits were analysed, and CFS assessments were performed in 47%. The completion rate exceeded 50% for patients over the age of 80. Patients with low triage priority were assessed to a low degree (24%). There was a diurnal variation with the highest completion rates seen for arrivals between 6 and 12 a.m. (58%). The survey response rate was 48%. The respondents rated the perceived relevance and the ease of use of the CFS with a median of 5 (IQR 2) on a scale with 7 being the highest. High workload, forgetfulness and critical illness were ranked as the top three barriers to assessment. The qualitative analysis showed that CFS assessments benefit from a clear routine and a sense of apparent relevance to emergency care.

**Conclusion:**

Most emergency staff perceived CFS as relevant and easy to use, yet far from all older ED patients were assessed. The most common barrier to assessment was high workload. Measures to facilitate use may include clarifying the purpose of the assessment with explicit follow-up actions, as well as formulating a clear routine for the assessment.

**Registration:**

The study was registered on ClinicalTrials.gov 2021-06-18 (identifier: NCT04931472).

**Supplementary Information:**

The online version contains supplementary material available at 10.1186/s12873-023-00894-8.

## Background

Frailty assessment of older patients presenting to the Emergency Department (ED) may help to estimate the risk of adverse events [1] and to deliver proper care [2]. Frailty has been shown to increase the risk of delirium, a serious alteration in cognition and attention, which has a significant association with morbidity and mortality [3]. The risk of delirium is also associated with length of stay in the ED [4] Therefore, it is crucial to the care given to older patients in the ED that frailty is identified, and that care is provided properly, to mitigate the risks for adverse events.

Implementation of frailty assessment has been advocated as part of emergency care [5, 6]. Research on frailty assessment tools in the ED has grown in recent years, and some tools have been introduced in clinical practice [7, 8]. Yet, the feasibility of using frailty screening tools in an ED setting is not fully understood [9]. For a tool to ultimately add value to patient care, it must be feasible to use in the intended context. For this reason, knowledge of feasibility is crucial for decisions and planning concerning a broader introduction [10].

The Clinical frailty scale (CFS) is a frailty assessment tool that has been introduced to some ED settings, for example in the United Kingdom [7]. The CFS is a judgement based 9-point scale (1-9) and was originally developed within the Canadian Study of Health and Aging [11]. It is made up of pictograms combined with clinical descriptions to help assign scores based on the assessment of a person’s function in daily life and cognitive status [12]. The tool has been evaluated for validity and reliability in the ED setting [13, 14], which together with its simplicity and the rapid deployment [15, 16] of approximately one minute [17, 18] has led to its recommended use in an ED setting.

However, our current understanding of how CFS is used in ED clinical practice remains limited. Tools can be both valid and reliable, but they will be of no benefit for patients if they are not used in clinical practice as intended. Some studies have reported the feasibility of applying CFS on older patients in the ED. Emergency department staff from the United Kingdom have reported feasibility measures in terms of experiences; that the CFS was easy to use on vignette cases [17], and that they feel confident using the tool in clinical practice [19]. Another way to assess feasibility is the completion rate, which in this context is defined as the proportion of completed CFS assessments to the total number of ED visits made by older people. Completion rates for CFS assessments have been investigated in Europe with reported levels as high as 98.9% [20] and 96.0% [21] when study personnel completed the assessments, but with results around 50% when the assessments were made during clinical work [7, 19]. From an implementation perspective, the need thus remains to identify which factors influence the use of CFS in the ED when it is performed as part of standard care [10, 22]. Therefore, this study aims to evaluate the feasibility of CFS in a standard care ED-setting by investigating how the CFS is used during clinical work, in combination with exploring ED staff’s experiences of using it.

This study had two approaches: “investigate completion rate” and “understand staff experiences”. To investigate completion rates, the following research question was shaped: 1)What is the overall completion rate of CFS assessments among ED staff, and how is this affected in relation to patient- and organisation related factors (e.g., age; triage priority; day of week and time of day)? To explore staff experiences, the questions were; 2)What are the ED staff’s experiences with: relevance; ease of use; time consumption; barriers and facilitators when using CFS in clinical practice? and; 3)What are the ED staff’s experiences of relevance of frailty assessment (not specifically with CFS) in the ED?

## Methods

### Study design and setting

This was a prospective mixed methods study carried out in three Swedish EDs, all located within the same region and organisation (Region Östergötland). The combination of an observational and survey design with analysis of both quantitative and qualitative data was chosen for the possibility of obtaining both a broader and deeper understanding of the research questions [23]. The study was approved by the Swedish Ethical Review Authority (permit 2021 − 00875) and registered on ClinicalTrials.gov (identifier: NCT04931472).

The characteristics for the three EDs and their respective recruitment periods are specified in Table 1: one University Hospital (UH), which also is the regional trauma centre and one of few EDs in Sweden run exclusively by emergency physicians, one community hospital ED (CH 1), and one rural community hospital ED (CH 2). None of the EDs operate a specific pathway or unit for older people.

**Table 1:** Characteristics and recruitment periods for the three participating EDs

	UH	CH 1	CH 2
**Annual ED visits**	50 000	50 000	25 000
**Type**	University Hospital	Urban Community Hospital	Rural Community Hospital
**Data collection periods**	- Demand: May/June 2021 - Acceptability: June-July 2021	- Demand: October/November 2021 - Acceptability: November/December 2021	- Demand: October/November 2021 - Acceptability: November/December 2021

Swedish emergency care organisations typically involve physicians (either emergency physicians or interns/residents from the specialties internal medicine; surgery; or orthopaedics), registered nurses and assistant nurses, all with varying experience [24]. There are no national guidelines regarding frailty assessment for Swedish EDs. However, a number of EDs assess frailty in some way, and of those using an established tool the CFS is the most common [8].

At the time for data collection, the CFS had recently been introduced as a clinical routine in the participating EDs but none of the institutions had established a system for monitoring the outcomes of the assessments. Prior to its introduction, all staff members were encouraged to participate in an e-learning course on the use of CFS. The content of the e-learning course was derived from the online training module developed by AIMS research group of Ottawa Hospital, Canada [25]. Three clinical vignettes were included along with the basic theoretical concept of frailty and its consequences for the patients’ health and functional aspects. Approximately 30 min were required to complete the course. Among ED staff, the completion rate for the e-learning course was 77%. Physicians employed elsewhere were not asked to complete the course but may still have performed it as the education was open to everyone in the organisation.

### Methods of measurements

Data for this study were collected in two phases:

1. Data about patient- and organisational related factors for patients with and without a CFS-assessment were collected over a period of six weeks. The start of the collection periods differed between the hospitals as the e-learning course could be completed at slightly different times, and we wanted to ensure the same conditions for all hospitals before data collection began. Data collection was performed at all hours during the study period. The team members who were responsible for the patient (typically a physician, registered nurse, and assistant nurse) were instructed that someone on the team should assess the patient during the ED visit. As the study was undertaken in a clinical context, the staff had access to standard clinical information such as the patient, relative/caregiver, and notes from the electronic medical records (EMR). We used the Swedish version of the Clinical Frailty Scale (CFS-9) [26], the CFS score was registered in a worksheet attached to the patients’ ED records.
2. Immediately after completion of the first phase, the ED staff received a survey (Webropol version 3, Webropol Oy, Helsinki, Finland) by email, with two reminders in the absence of a response within 14 days. The survey investigated staff experiences and was developed specifically for this study. We selected previously studied acceptability areas [17, 18, 27, 28] of relevance to the current study. The recommendations made by Statistics Sweden [29] for fundamental elements like wording, questioning style, and response options served as our guide during the survey’s structuring. To confirm content validity, it was pre-tested by five persons (clinicians and non-health professionals) who were interviewed about the perceived meaning of the questions. This generated some adjustments to improve clarity. The survey (available as supplemental material) consisted of Likert scales, multiple-choice questions, and open textboxes.

### Inclusion of participants

For phase one, informed consent was waived by the Ethical Review Authority. For phase two, staff provided their consent by answering the survey. All physicians, registered nurses, and assistant nurses who had been working clinically in the EDs during phase one were invited to anonymously answer the survey.

### Outcomes and data analysis

Bowen et al. [10] present eight general focus-areas suitable for feasibility studies, of which the two areas “demand” (frequency of assessment and patterns of use) and “acceptability” (user satisfaction, barriers, and facilitators) are investigated in this study. For the area demand, we calculated the completion rate of CFS-assessments, both overall and relative to patient-related factors and organisational factors. Acceptability was examined by investigating the staff’s experience of relevance, ease of use, time consumption, barriers to use, facilitators to use, and the importance of frailty assessment in general. The data sources and analyses for each outcome are further presented in Table 2. All statistics are descriptive and reported as frequencies, median and interquartile ranges (IQR), or as number and percentages (%). Significant analyses for categorical variables were calculated with the Chi2-test or Fisher’s exact test when the number of outcomes were lower than 5 in any group, and with independent samples median test for continuous measures like age. All statistical analysis was done in IBM SPSS Statistics for Windows, version 27.

**Table 2:** Description of the outcomes

	Feasibility outcomes	Data source	Data analysis
**Demand**	- Completion rate of CFS-assessments, overall - Completion rate relative to patient-related factors - Completion rate relative to organisation factors	- Worksheet: CFS-assessments - EMR: age; sex; mode of arrival; triage priority; discharge destination; day of week and time of day	Descriptive statistics
**Acceptability**	- Staff’s perception of relevance - Staff’s perception of ease of use - Staff’s perception of time consumption - Staff’s perception of barriers to use - Staff’s perception of facilitators to use - Staff’s perception of importance of frailty assessment in general	- Survey answered by ED staff (physicians, registered nurses, and assistant nurses)	Descriptive statistics of quantitative data, and qualitative content analysis of comments in the open textboxes

In addition, we performed a conventional qualitative content analysis according to Hsieh and Shannon [30] to outline the meanings of the comments in the open textboxes. The analysis was performed by two authors (EH and SME), and the first author (EH) led the analysis. EH has previous experience with the method. Both researchers have also completed basic and advanced courses in qualitative methodology. The pre-understanding for both the authors consisted of a specific interest in the condition of frailty, and in addition many years of experience of emergency care. The analysis thus began with the personal pre-understanding being written down and reflected on; this was further repeated during the analysis process. The data were treated as a uniform text and read through repeatedly to obtain a sense of the whole. All sentences related to the research questions were first marked as “interesting” and then coded with a label describing their meaning. The coded sentences were organised into categories and subcategories (Table 3). The authors repeatedly returned to the text, both individually and jointly, to understand what the participants were communicating. The results were continuously discussed by the two authors and revised until an agreement was reached. The analysis involved a certain degree of interpretation, but the purpose of the study and the nature of the data resulted in a manifest analysis.

**Table 3:** Example of the qualitative analysis process

Meanings marked with the label “No need for specific tool”	Subcategory	Category
However, I believe that evaluation of frailty is already included in the medical assessment and have difficulty seeing how a score would change my handling.	Prefer clinical judgement	Lack of motivation
Anyone with a medical or nursing education should automatically be able to assess frailty in a patient without the use of CFS.		
I request the information in the assessment anyway, without making an estimate according to a specific tool.		

## Results

**Fig. 1:**
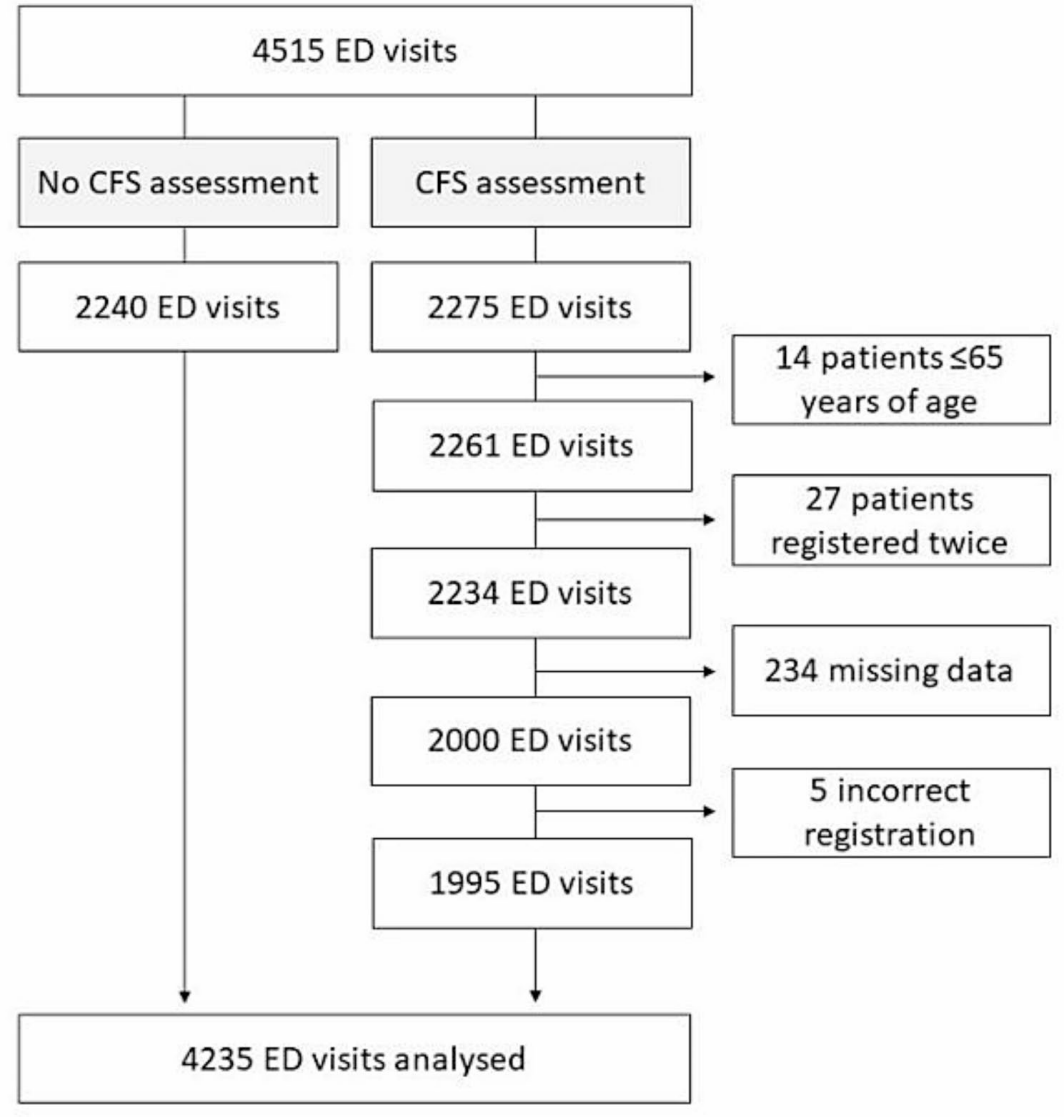
Flow chart describing the inclusion process for ED visits made by patients ≥ 65 years old

A total of 4515 ED visits by patients ≥ 65 years were made during the data collection period. Of these, 280 were excluded, mostly due to missing data. There were 1995 visits with completed assessments and 2240 non-assessed visits, which together comprise the sample of 4235 visits analysed in this study (Fig. 1).

### Demand

The overall completion rate of CFS-based assessments was 47.0%. The completion rate increased with the age of the patients, and for the oldest (≥ 96 years of age) it was 76.9%. The completion rate exceeded 50% for those who arrived by ambulance (56.3%) or by recumbent transport (63.2%). Patients with triage priorities 2 (very urgent) and 3 (urgent) had CFS completion rates of just over 50%, while patients with minor injuries had the lowest proportion of completed assessments (24.0%) (Table 4). There were minor differences in completion rates between the days of the week, with the lowest on Sundays (43.5%). During the day, the completion rate was highest (58.1%) for patients who arrived at the ED between 06:00 and 12:00 am.

**Table 4:** The completion rate of CFS-assessments in relation to patient- and organisation related factors

	Total	Patients with CFS assessment	Patients without CFS assessment	P-value
Number, n (%)	4235	1995 (47.1)	2240 (52.9)	
Women, n (%)	2234 (52.8)	1091 (54.7)	1143 (51.0)	
Age, median (IQR)	77 (12)	78 (12)	76 (11)	0.000
**Age, five years strata**				
65–70, n (%)	882	331 (37.5)	551 (62.5)	0.000
71–75, n (%)	925	423 (45.7)	502 (54.3)	0.009
76–80, n (%)	902	415 (46.0)	487 (54.0)	0.017
81–85, n (%)	740	373 (50.4)	367 (49.6)	0.825
86–90, n (%)	500	283 (56.6)	217 (43.4)	0.003
91–95, n (%)	234	130 (55.6)	104 (44.4)	0.089
96-, n (%)	52	40 (76.9)	12 (23.1)	0.000
**Triage priority at arrival**				
1 (immediate), n (%)	275	115 (41.8)	160 (58.2)	0.007
2 (very urgent), n (%)	1233	632 (51.3)	601 (48.7)	0.377
3 (urgent), n (%)	1901	973 (51.2)	928 (48.8)	0.302
4 (non-urgent), n (%)	511	217 (42.5)	294 (57.5)	0.001
5 (minor injuries), n (%)	229	55 (24.0)	174 (76.0)	0.000
Missing, n (%)	86	3 (3.5)	83 (96.5)	
**Mode of arrival)**				
Walk in, n (%)	2221	880 (39.6)	1341 (60.4)	0.000
Ambulance, n (%)	1891	1065 (56.3)	826 (43.7)	0.000
Recumbent patient transport, n (%)	38	24 (63.2)	14 (36.8)	0.105
Missing or other, n (%)	85	26 (30.6)	59 (69.4)	
**Discharge destination**				
Home, n (%)	2447	1104 (45.1)	1343 (54.9)	0.000
Admitted, n (%)	1679	879 (52.4)	800 (47.6)	0.054
Primary care, n (%)	58	7 (12.1)	51 (87.9)	0.000
Left without being seen, n (%)	39	5 (12.8)	34 (87.2)	0.000
Deceased in the ED, n (%)	12	0 (0.0)	12 (100.0)	0.000
**Day of arrival**				
Monday, n (%)	705	338 (47.9)	367 (52.1)	0.275
Tuesday, n (%)	590	300 (50.8)	290 (49.2)	0.681
Wednesday, n (%)	601	299 (49.8)	302 (50.2)	0.903
Thursday, n (%)	611	271 (44.4)	340 (55.6)	0.005
Friday, n (%)	644	287 (44.6)	357 (55.4)	0.006
Saturday, n (%)	542	264 (48.7)	278 (51.3)	0.548
Sunday, n (%)	542	236 (43.5)	306 (56.5)	0.003
**Time of arrival**				
24.00–06.00, n (%)	336	141 (42.0)	195 (58.0)	0.003
06.01-12.00, n (%)	1214	705 (58.1)	509 (41.9)	0.000
12.01-18.00, n (%)	1840	826 (44.9)	1014 (55.1)	0.000
18.01–23.59, n (%)	845	323 (38.2)	522 (61.8)	0.000

### Acceptability

In total, 475 ED staff (216 physicians, 148 registered nurses, and 111 assistant nurses) received the survey on perceived user satisfaction, barriers, and facilitators, and 229 (48.0%) responded. Eight declined participation, leaving 221 answers to analyse. The number of respondents was similar between professions, with 78 physicians (divided into 50 emergency physicians, and 28 interns/residents from other specialties), 73 registered nurses, and 70 assistant nurses. The distribution of the percentage of respondents between the hospitals was: UH, 48.9%; CH 1, 28.5% and; CH2, 22.6%. Most participants (70.0%) were women; the all-over median age was 35 years (IQR 17), and median work experience was 8 years (IQR 12).

Information on perceived user satisfaction, barriers, and facilitators was collected using a 7-point Likert scale. Most respondents had positive experiences with the relevance of CFS assessments, relevance to frailty assessments in general, ease of use of the CFS, and perceived time required for CFS assessments (Table 5).

**Table 5:** Emergency department staff´s responses to their experiences of using the CFS

Variable	Responses, n	Results, median (IQR)
CFS, relevance	221	5 (2)
Frailty assessment in general, relevance	221	6 (2)
CFS, ease of use	178	5 (2)
CFS, time consumption	178	3 (2)

**Fig. 2:**
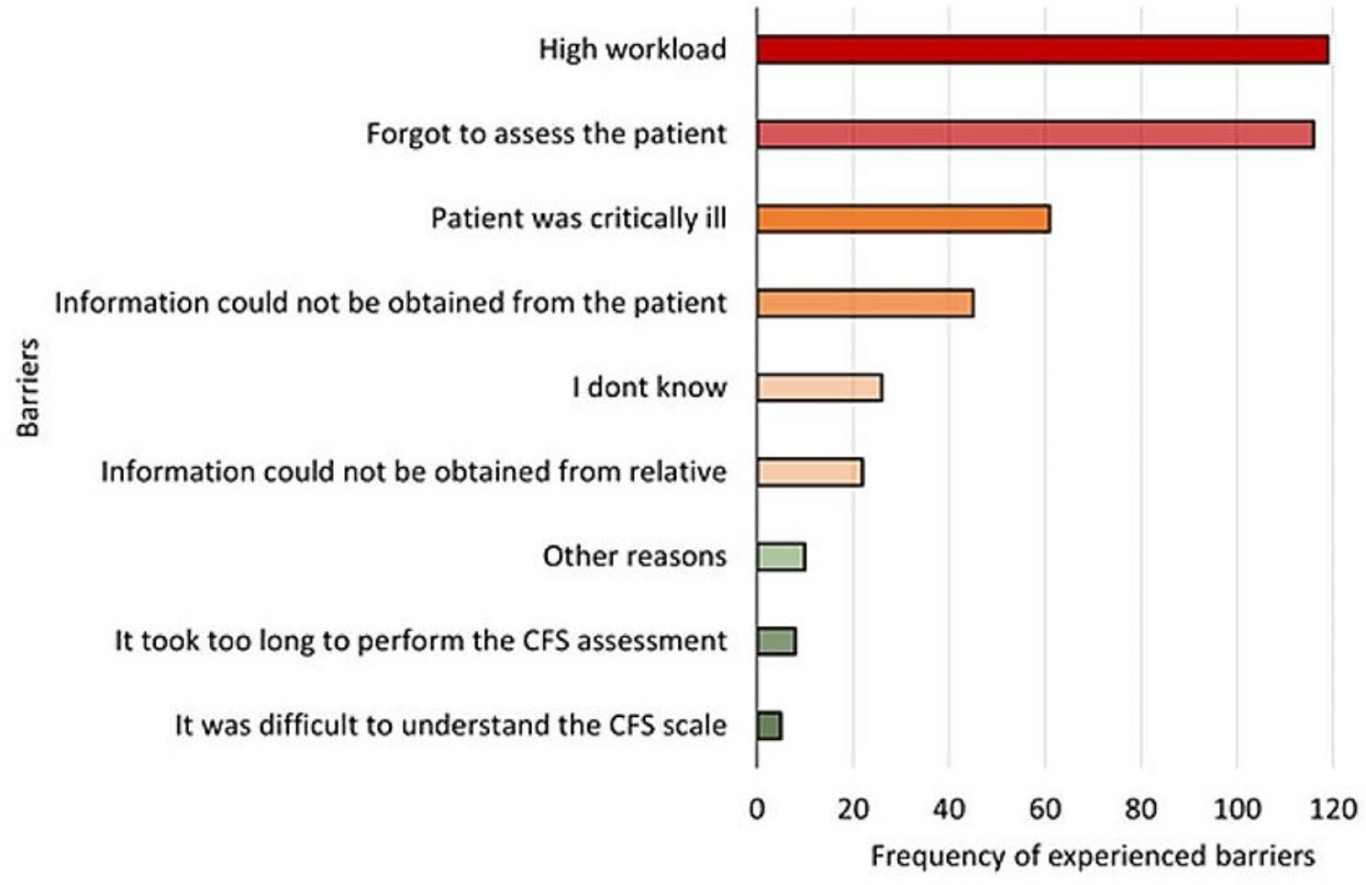
The frequency of ED staff reported barriers to assess patients with CFS. The number of times each barrier was selected. Participants could select all available options that they perceived as a barrier to CFS assessment. The question was answered by 209 respondents

To identify barriers to using the CFS, we asked: “In cases where you did not assess patients ≥ 65 years of age, what was the reason?”. Participants could select one or more predefined answers, as well as provide free comments regarding other barriers to CFS assessment (Fig. 2). High workload and forgetfulness were the most frequently selected barriers to assessing the patient with CFS, while difficulty understanding the scale or time-consuming assessment were the least reported barriers.

### Qualitative analysis

**Fig. 3:**
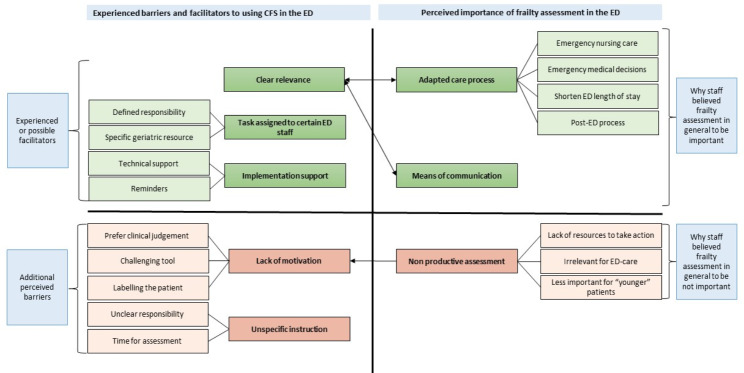
The result of the qualitative analysis: the eight categories and 16 subcategories

The three open text boxes yielded 194 comments, written by 124 unique ED staff, divided between 41 physicians, 48 registered nurses, and 35 assistant nurses. The questions and subsequent comments were about: additional perceived barriers to CFS assessment; existing or potential facilitators; and perceived importance of identifying frailty (in general) in the ED. The analysis resulted in a total of eight categories and 16 subcategories. The categories and subcategories are illustrated in Fig. 3 and presented in more detail below. Illustrative quotes are marked with profession (Physician = Ph, Registered nurse = RN and Assistant nurse = AN) and hospital (University hospital = UH, Community hospital = CH 1 or CH 2).

### Additional barriers to CFS-assessment

#### Unspecific instruction

The description that assessments were not made because the respondents did not feel responsible for it, resulted in the subcategory “Unclear responsibility”. The instruction to the staff was that someone on the team should do the CFS assessment during the patient’s stay in the ED. It may be that the imprecise wording contributed to fewer assessments being made. The subcategory “Time for assessment” was formed by respondents’ descriptions of the fact that the assessments were postponed, which resulted in the patient being discharged without the assessment being carried out.

*“…as well as when a patient had been quickly discharged home or to a ward” (AN, CH 1)*.

#### Lack of motivation

The subcategory “Preferring clinical judgement” stemmed from the view that the use of a specific assessment tool is unnecessary, as frailty can be assessed using clinical judgement alone. The experience that the use of CFS is challenging was partly based on the need to understand the patient’s usual ability to cope with daily activities, and partly on the difficulty of knowing which questions to ask. This was framed in the subcategory “Challenging Tool”.

*“Assessment of frailty is important, but CFS is challenging as there are many questions about the patients´ everyday life…difficult to evaluate the steps in the assessment…” (RN, CH 1)*.

The third subcategory “Labelling the patient” involves the experience of labelling the patient in a definitive way by grading the person on a scale.

*“It feels as if I put a stamp on the patient…that the assessment is definitive in some way” (RN, UH)*.

### Existing or potential facilitators for CFS-assessment

#### Clear relevance

The category “Clear relevance” was based on the expressions that it was facilitating to maintain, or obtain a sense that a CFS assessment leads to something significant for the patient.

*“To continue having the feeling that it is something significant and worth making time for” (Ph, UH)*.

#### Tasks assigned to certain ED staff

Another facilitator is described in the subcategory “Defined responsibility”, and would be to dedicate the assessment to specific personnel. Either within each care team (e.g., the registered nurse is always responsible) or at a specific position (e.g. triage). The proposal in the subcategory “Specific geriatric resource” concerned an exclusive geriatric resource that operates on all care teams.

*“Assessment could perhaps take place during triage so that it is always done; standardisation often improves this sort of procedures” (Ph, CH 1)*.

#### Implementation support

It was further described that continued and additional implementation support would facilitate assessments. Suggestions for verbal or visual reminders formed the “Reminders” subcategory, while integrating the CFS into the EMR formed the “Technical Support” subcategory.

### Perceived importance of frailty assessment (in general) in the ED

The perceived importance of frailty assessment in general interacts to a certain degree with the experience of barriers and facilitators. This is illustrated in Fig. 3.

#### Adapted care process

Those who considered frailty assessment to be important in the ED described it as providing significant information about the patient, leading to an adapted care process. The subcategory “Emergency nursing care” included adapted nursing interventions in the ED. Examples given were increased attention to nutritional and elimination needs; position changes; “real” beds and; more frequent nursing rounds.

*“Many older people with frailty are in greater need of nursing rounds as they can rapidly deteriorate, as a result of decreased reserves” (RN, CH 2)*.

In the subcategory “Emergency medical decisions” the importance of taking frailty into account in medical reasoning was described. Frailty was considered to influence the acute illness, and decisions about: drug treatment; the planned content of care and whether the patient should be admitted to hospital or discharged home.

*“…to be extra attentive and think broadly due to underlying frailty” (Ph, CH 2)*.

Within the subcategory “Shorten the ED length of stay”, the risk of patient harm during waiting times was commented on, as was the importance of rapid care processes for patients living with frailty. Comments in the subcategory “Post-ED process” showed identification of frailty in the ED being considered to influence care, or support, after the emergency visit.

### Means of communication

This category involves expressions that frailty assessment is a way to a concordant view of the concept and thus facilitate communication when discussing the patient on the team or at hand-off, but also as a means to follow the degree of frailty over time.

### Non-productive assessment

Informants who stated that frailty assessment was unimportant expressed that it did not affect the care provided. In the subcategory “Lack of resources to take action”, respondents voiced a lack of either time or personnel to act on the obtained information. In the subcategory “Irrelevant to emergency care” the perspective was that the information about frailty was of no use in emergency care.

*“The patients are here for a short time, and I don’t think that it (frailty assessment) helps the work” (AN, CH 1)*.

The subcategory “Less important for younger patients” included experiences of that 65 years was too low an age limit, as many people of that age are still fit.

## Discussion

The recommendation to identify frailty in older patients in acute care settings, is well-founded in extensive literature [31]. In comparison to traditional triage systems, CFS can effectively identify the risk of adverse outcomes in older individuals [32, 33]. Therefore, with this study, we aimed to understand how the CFS is used in a standard care ED setting, and what constitutes barriers and facilitators to using it. We found an overall completion rate of 47%, and most responders reported a positive attitude towards the CFS regarding its relevance, ease of use and time consumption. The qualitative analysis contributed to a better understanding of what can influence the motivation for frailty assessment.

### Demand

Regarding completion rate, results similar to ours have been reported for both CFS [19] and other frailty assessment tools [9]. A recent national survey in the UK [7] found a mean compliance rate of 50% for frailty assessment, but with a significant variation of 2.2–100%. McGrath et al. [19] reported an overall completion rate of 47%, although 73% for patients arriving by ambulance. These findings resonate well with our data, since staff were more likely to assess patients who arrived by ambulance and patients who belonged to the older age groups. These factors have previously been shown to be related, with patients over the age of 80 being the group most often arriving by ambulance [34].

The proportion of CFS assessments was mainly consistent with the daily pattern of occupancy in the ED [35], i.e., a lower proportion of patients were assessed with CFS during hours when workload is at its highest. Therefore, if an ED could allocate specific geriatric resources, as suggested as one of the facilitators in this study, it would likely be most beneficial during periods of peak workload. However, in this study, completion rates remained low even after midnight, indicating that other factors, in addition to workload, may be influencing the rates, especially as patient occupancy gradually decreases during the night [35].

The findings of this study, as well as previous reports, raise the question of what constitutes an acceptable completion rate? As emergency care staff find themselves in an environment characterised by fast pace and sometimes with a workload exceeding the available resources, reasoning is required about when and how frailty assessment is most effectively performed. One of the opinions expressed in this study was that 65 years of age is too young to serve as the cut-off for frailty assessment. Still, the potential benefit of a frailty assessment is to identify increased risk of adverse events regardless of chronological age [36], which would then be lost for many patients if too high an upper age limit was applied. Based on the age difference between the groups “patients with CFS-assessments” and “patients without CFS-assessments” (median 78 versus 76), one could hypothesise that staff in the present study prioritised assessing patients of older age because they are more likely to live with frailty. Alternatively, staff simply used their clinical judgement as a “first check”, before prioritising to use the CFS. Previous reports indicate that clinical judgement can be more sensitive but less specific to frailty, than both the CFS [14] and a Dutch tool screening for vulnerability for 1-year mortality (Veiligheids Management Systeem) [37]. It would be worthwhile to assess the accuracy of an approach where ED staff first rely on their clinical judgement to identify potential frailty and then conduct a standardised CFS assessment, primarily for patients suspected of having frailty.

### Acceptability

#### Relevance

Respondents in the current study rated the relevance of CFS in the ED at a median of five on a seven-point scale. Further, like in the study by Liu et al. [28], our qualitative analysis indicated that some staff adapted both nursing and medical management for patients living with frailty, and that whether a person was living with frailty or not was seen as important knowledge for adapting the continued care process to the person’s needs. Although the low response rate precludes any definite conclusions, the results still suggest that the information obtained by CFS assessment has the potential to contribute to a more person-centred care in an ED-setting.

However, our analysis also showed that frailty assessment was perceived as irrelevant by the staff due to perceived lack of impact on care. Similar results of ED staff questioning the usefulness of frailty assessment have previously been reported [27, 28, 38]. The lack of precise knowledge about the effect of interventions for patients living with frailty may be a contributing factor to the experience that frailty assessment does not add value to patient care in the ED. Interventions such as geriatric assessment, discharge management and post discharge follow-up have been evaluated in an ED context, but with inconsistent results [39]. Based on the results from this and previous studies, and on our own experience from clinical ED settings, we believe that perceived relevance is a factor with major impact on the propensity to perform frailty assessments. Thus, going forward, significant efforts should be made to evaluate various ED-based interventions for older people living with frailty, as the triggering of effective interventions will most likely reduce the feeling among ED staff of a non-productive assessment.

#### Ease of use

CFS was rated as easy to use by the respondents (median 5 on a scale of 7), which is consistent with results from Elliot et al. [17] where CFS also scored well after being used on vignette cases. Nonetheless, it is likely a topic that should be revisited during implementation, as this study demonstrates that the CFS can be perceived as challenging, with difficulty posing the right questions. Perhaps a lack of self-perceived competence contributes to uncertainty and leads to the experienced barrier of “labelling the patient”. The concern for categorising patients through frailty assessment has previously been described [17, 38] and highlights the need for continuous ethical reflection and competence development regarding this complex patient group. As part of the training, the classification tree based on the CFS levels, developed by Theou et al. [40] may be used to assist in asking questions.

#### Barriers

This study identified “high workload,“ “forgetfulness,“ and “critically ill patient” as the perceived top three barriers to CFS assessment, all of which have been described as impediments to frailty screening [17, 27]. In addition, it was also voiced that an unspecific instruction had contributed to missed CFS assessments. In line with this, respondents expressed that clear instructions would facilitate. Conditions naturally vary among different EDs, but the result of this study suggests the completion rate would probably benefit from specifying the role/profession responsible for the assessment and/or the timing during the ED visit for when to do the assessment.

#### Strengths and limitations

This multicentre study’s primary strength lies in its inclusion of personnel from EDs of varying sizes, organisational structures, and medical staffing, providing a diverse perspective. Additionally, the study conducted a demand analysis on a substantial number of patients, and its mixed-method design further adds to its robustness. While the detail and depth of data from open-text comments may be somewhat limited compared to data obtained from interviews, we contend that the experiences shared by a large number of participants serve the study’s intended purpose. The breadth and descriptive nature of the results offer valuable insights for future research and clinical development in a relatively unexplored area. To enhance credibility, despite the inability to ask clarifying questions to anonymous respondents, the qualitative analysis involved two authors who diligently revisited the data to clarify its meaning.

Our study also had limitations. The CFS had recently been introduced into the participating EDs which may negatively affect the generalisability. Further, the study would have been enhanced if we had recorded which staff members conducted the assessments, allowing us to compare the profiles of CFS assessors and non-CFS assessors. The survey was designed for the current study, and even though it was tested and adjusted before its use, this may have affected the validity of the results. The survey was designed to be anonymous so that employees would feel comfortable answering it, hopefully resulting in a high response rate. However, the response rate was just under 50%, which may have biased the results, as it is possible that individuals who were explicitly positive or negative to frailty screening responded more frequently than others. Although the pre-understanding was reflected on through the analysis process, it cannot be ruled out that our professional roles as ED care providers biased the synthesis of the results in the qualitative analysis. Finally, we aimed to study the time consumption for CFS assessment and therefore measured the time needed for completing the worksheet. However, as this only measures time taken to complete the paperwork rather than the actual time required for assessment, the data was excluded.

## Conclusion

This study implies that the CFS shows potential for being effectively implemented in an ED setting because most participants found it relevant and easy to use. Yet, far from all older ED patients were assessed with CFS which points to challenges for implementation in clinical practice. The most common barrier to assessment was high workload. Measures to facilitate use may include clarifying the purpose of the assessment with explicit follow-up actions, as well as formulating a clear routine for the assessment.

### Electronic supplementary material

Below is the link to the electronic supplementary material.

Supplementary Material 1

## Data Availability

The datasets used and/or analysed during the current study are available from the corresponding author on reasonable request.

## Abbreviations

CFS: Clinical Frailty Scale
ED: Emergency department
UH: University hospital
CH: Community hospital
EMR: Electronic medical records
Ph: Physician
RN: Registered nurse
AN: Assistant nurse
