# Supplementary material for: Is the clinical frailty scale feasible to use in an emergency department setting? A mixed methods study
Source: BMC Emerg Med. 2023 Oct 26;23:124. doi: 10.1186/s12873-023-00894-8 (PMC10601295; doi:10.1186/s12873-023-00894-8)
Supplement: Supplementary file 1 — Supplementary Material 1 [file 12873_2023_894_MOESM1_ESM.pdf]

.

Feasibility and acceptability of the Clinical Frailty Scale in a Swedish Emergency Department setting

Survey

Usefulness of the Clinical Frailty Scale (CFS) and Loss of Independence (LoI) in a Swedish emergency department

Mandatory fields are marked with an asterisk (*) and must be filled in to complete the form.

Request for participation in the research project "Evaluation of instrument for frailty assessment at Swedish emergency departments "

The survey is sent to all physicians, nurses, and assistant nurses who during clinical work has assessed patients at the emergency department US, ViN or LiM with CFS / LoI during the time for data collection for the research project.

One of the purposes of the project is to find out about if these tools are applicable to an emergency department in Sweden, and what facilitates or complicates their use. This request concerns participation in a survey that helps us answering those questions. The survey is about your experience of using CFS / LoI in clinical work, what hindered its use and what may facilitate the use.

You participate in the study by answering this survey, it takes about 7 minutes to fill in. Your answers are anonymous and cannot be traced to you either by the research group or in the finished material. The results will be presented in a scientific journal, but also at each emergency department as this also evaluates a joint quality work (implementation of CFS).

Your participation is voluntary, and you can cancel the survey at any time before submitting your answers. As the answers are anonymous even for the researchers, replies will not be able to be revoked after submission.

If you want to know more about the study, you are welcome to contact Erika Hörlin (erika.horlin@regionostergotland.se), Samia Munir Ehrlington (samia.munir.ehrlington@regionostergotland.se) or Rani Toll (rani.toll.john@regionostergotland.se). If you decide to respond to the online survey, you agree to participate the study. *

I agree to answer the survey and participate in the research project "Evaluation of instrument for frailty assessment at Swedish emergency department "

I do not want to answer the survey/participate in the research project

---

1a. What is your profession?

Physician

Registered nurse

Nurse assistant

1b. (If "Physician" is chosen) Which is your position?

Emergency physician (Resident or Specialist)

Resident physician from another unit

Intern

Other

2. What is your gender?

Male

Female

Other

3. What is your age?
(18--70)

4. For how many years have you been working in your profession (since graduation)?
(0--40)

---

5. You have been working clinically during the period for data collection for the evaluation of CFS and LoI. Have you, or someone on your team been assessing patients with CFS/LoI? *

Yes I have personally assessed one or more patients with CFS and/or LOI

I have not done the assessment in person, but someone else on the team have done it

No assessment with CFS and/or LOI has been made on any of the team's patients when I have been working

I don´t know

6. How many patients do you estimate that you have assessed with CFS/LoI?

1-10

11-20

21-30

1-40

41-50

>50

7. If you had 10 patients ≥65 years on your team during the time for data collection: how many of these did you rate (on average) with CFS/LoI?

0

1

2

3

4

5

6

7

8

9

10

8. In cases where you did not assess patients ≥65 years, what was the reason? (multiple options are possible)

The information required for an assessment could not be obtained from the patient

The information required for an assessment could not be obtained from relatives

The patient was too ill

Assessment with CFS could not be prioritised due to high workload

Assessment with LoI could not be prioritised due to high workload

The assessments (CFS) took too long

The assessments (LoI) took too long

CFS was difficult to understand

LOI was difficult to understand

We forgot to assess the patient

Other reason: (text box)

I don´t know

9. What would have made it easier for you and enabled an assessment of the patient in those cases you did not make assessments with CFS and/or LOI on patients over 65 years of age? Please write down your thoughts about this in the box below.

(text box)

____

10. What is your experience of determining a person's CFS score? Grade between 1 (Not at all easy) and 7 (Very easy)

1 Not at all easy

2

3

4

5

6

7 Very easy

11. How time-consuming do you perceive it to be, to assess a person with CFS? Grade between 1 (Not at all time consuming) and 7 (Very time consuming) *

1 Not at all time consuming

2

3

4

5

6

7 Very time consuming

12. How relevant do you think CFS is to use in the Emergency Department? Grade between 1 (Completely irrelevant) and 7 (Very relevant)

1 Completely irrelevant

2

3

4

5

6

7 Very relevant

I don’t know

13. What is your experience of determining a person's LoI status? Grade between 1 (Not at all easy) and 7 (Very easy)

1 Not at all easy

2

3

4

5

6

7 Very easy

14. How time-consuming do you perceive it to be, to assess a person with LoI? Grade between 1 (Not at all time consuming) and 7 (Very time consuming) *

1 Not at all time consuming

2

3

4

5

6

7 Very time consuming

15. How relevant do you think LoI is to use in the Emergency Department? Grade between 1 (Completely irrelevant) and 7 (Very relevant)

1 Completely irrelevant

2

3

4

5

6

7 Very relevant

I don’t know

---

16. How important or unimportant do you believe it is to identify frailty in patients ≥65 years of age in the emergency department? Grade between 1 (Completely unimportant) - 7 (Very important)

1 Completely unimportant

2

3

4

5

6

7 Very important

I don´t know

17. Please describe why you think it is important/unimportant to identify frailty in the emergency department

(Text box)

18. How motivated are you to assess people who are ≥65 years for frailty in the emergency department? Rate between 1 (Completely unmotivated) to 7 (Very motivated)

1 Completely unmotivated

2

3

4

5

6

7 Very motivated

I don´t know

Thank You!
